# Physical Restraint Use in Swiss Nursing Homes: Two New National Quality Indicators

**DOI:** 10.1093/geroni/igaa057.2300

**Published:** 2020-12-16

**Authors:** Lauriane Favez, Franziska Zúñiga, Catherine Blatter, Narayan Sharma, Michael Simon

**Affiliations:** 1 University of Basel, Basel, Basel-Stadt, Switzerland; 2 Basel University, Basel, Basel-Stadt, Switzerland

## Abstract

Quality indicators are used in nursing homes to assess physical restraint use. Switzerland introduced two publicly reported indicators measuring the use of 1) bedrails and 2) trunk fixation or seating that prevent standing up. Whether these indicators show good between-provider variability is unknown. The study aimed to measure the prevalence of physical restraint use and assess their between-provider variability using a cross-sectional, multicentre study of a convenience sample of nursing homes. The between-provider variability of the indicators was assessed with intraclass correlation 1 and with caterpillar plots based on Empirical Bayes estimates. We included 11,412 residents from 152 nursing homes. Prevalence rates were 13.5% (n=1’433) for bedrails and 3.6% (n=411) for trunk fixation / seating that prevent standing up. For the first indicator, intraclass correlation 1 was 0.245 (95%-CI 0.197-0.286), for the second 0.343 (95%-CI 0.235-0.405). The two indicators showed good between-provider variability and can be recommended for public reporting. Part of a symposium sponsored by Systems Research in Long-Term Care Interest Group.

